# Lecture on Displacements of the Uterus

**Published:** 1839-05-25

**Authors:** W. Harris

**Affiliations:** At the Philadelphia Medical Institute


### CLINICAL LECTURE.

### LECTURE ON DISPLACEMENTS OF THE UTERUS, *delivered by* W. HARRIS, M. D., *at the Philadelphia Medical Institute.*

HAVING finished a minute anatomical description of the form, dimensions, and structure of the uterus, there can be no more appropriate place than this, to treat of the displacements of that organ in its unimpregnated state. Those which arise from gestation and parturition, may more properly belong to another part of the course.

PROLAPSUS UTERI.

Prolapsus uteri is the form of displacement which occurs most frequently, and when we consider the situation of the uterus, and its slight connection with the surrounding parts, we are not surprised at its common occurrence.

Females of lymphatic temperaments, and of relaxed muscular fibre, are predisposed to this displacement; also those who stand much, especially washer-women and shop-keepers; those who have had frequent accouchemens; those who sit up too soon after parturition, and those who have preternaturally large pelves.

Tight lacing with corsets, violent dancing or leaping, constipation, and difficult evacuations, will also cause this displacement.

Fluor-albus, in the opinion of Dr. Dewees, is one of the most common causes of prolapsus. He contends that the vagina is the principal support of the uterus, and that as this disease weakens the foundation, the superstructure must fall.

I unite in opinion with Dr. Dewees, that the vagina is the chief support of the uterus, but cannot agree with him that fluor-albus is the most common cause of prolapsus. On the contrary, my observations lead me to the conclusion that a majority of cases are brought on from sitting up too soon after delivery. Immediately after the birth of the child, the vagina is larger, and more relaxed than is natural; hence, if, in this state, the female sit up in an erect position, the uterus will be likely, from want of support, to fall down into the vagina.

I know, from experience, moreover, that long confinement to a horizontal position, after delivery, will cure the disease, if it had existed before the last pregnancy.

Mrs. H., aged thirty-five, of leucophlegmatic temperament, suffered great inconvenience from prolapsus uteri. In 1835 she became pregnant the fourth time, and during the last month of gestation her uterus rose above the superior strait, and she ceased to suffer from the prolapsus.

After the birth of her child, I gave it as my judgment, that if she would continue in a horizontal position from four to six weeks, without at any time, for a moment, assuming the erect position, it would cure her.

She submitted accordingly, and a complete cure was the consequence. She has never had the slightest return of the disease, although she has since had another child.

It may not be improper to remark, that during this lady's confinement to the horizontal position, two blocks of wood, each six inches thick, were placed under the foot-posts of the bedstead, which raised her hips, and, by gravitation, took the weight of the intestines off the fundus of the uterus, and the weight of itself off the vagina, and the lateral and round ligaments, until they all had time to recover their wonted tone. I attach much importance to this part of the treatment.

The degree of the descent of the uterus differs from a slight depression to a complete displacement. I was once called to the case of an intemperate woman, who had lain all night, in a state of inebriation, in the fence corner. Upon examination, I found that an inversion of the vagina had taken place, and that the whole uterus was projecting beyond the vulva; that it was of a livid colour, strangulated, and on the verge of mortification.

I cleansed and softened the protruding part with warm water, then anointed it with oil, after which I placed the os uteri against the palm of my hand, and, by a regular and steady pressure, was soon able to restore the organ to its proper situation, and to retain it there by an ordinary pessary.

When consulted by a female patient, the symptoms that would lead us to suspect that she had prolapsus uteri, are a sense of weight within the vagina and upon the perineum; a dragging sensation about the loins; a frequent desire to make water, and the urine disagreeably hot; tormina and tenesmus, and a sense of weight about the verge of the anus; and pain in the back, hips, and thighs, which sometimes is very distressing during long walks. If the above symptoms are all aggravated by walking, and all subside, or are greatly mitigated after remaining a few hours in a recumbent position, the physician may feel great, almost assured confidence, that she is labouring under procidentia uteri, but should never declare, positively, that this complaint is present without a careful per vaginam examination.

If the above indications of prolapsus be present, and, upon examination, in a horizontal position, the os uteri is not found resting upon the perineum, but high up, in its ordinary position, the physician should not rest satisfied until he has examined her in an erect position. It is sometimes, indeed, necessary to make the patient assume a squatting attitude, before the extent of the prolapsus can be fully ascertained.

A pessary is thought, by most authors, to be the only efficient remedy for this complaint. They have been constructed of various materials, such as sponges, cork, wax, caoutchouc, silver, gold, and glass. The last material is now generally preferred in this country, especially in Philadelphia. Some females, however, positively refuse to wear the glass pessary, lest by some fall, or blow, it might be broken within them, to their great injury. Such persons, for the most part, prefer the silver pessary gilded, which is, indeed, a very light and beautiful article. They are made, too, of various forms, and dimensions, such as a complete sphere, a spheroid, an ellipsis, a flattened ellipsis, &c.; but the shape now generally preferred is circular, having a convex surface below, and a concave surface above, with a hole in the centre. The object of the central aperture is to allow the secretions of the parts to escape.

Before introducing the pessary, the first time, the patient should have her bowels evacuated with a little castor oil, and remain in bed quietly for twenty-four hours, or longer, in order that all irritation of the parts may have time to subside.

The patient should be placed in bed in the same attitude recommended for the introduction of the catheter. The vulva and the instrument should both be freely anointed with hog's lard, or lubricated with flaxseed tea; the labia should be separated by the thumb on one side and the index finger upon the other; the circular edge of the pessary is then to be pressed against the posterior commissure of the vulva, directing the force backwards towards the internal face of the perineum, and then, by a slight rotatory motion, it will pass into the vagina, if it is not too large, without much difficulty. If it enter with difficulty, it may be facilitated by changing its direction, so as to cut the genital fissure a little obliquely. As soon as the instrument has completely passed through the os externum, with the index finger of the right hand, the operator should give it a transverse direction, being careful to place the concave surface upwards, and the os uteri over the central hole.

As soon as the instrument is properly adjusted, the patient should be directed to rise and walk about the room, that the operator may ascertain whether it adds to her comfort. If it is too large, it will give pain opposite the rectum or urethra; and if too small, it will escape.

We must be careful not to let her go to the privy for the first few days after it is introduced, as it sometimes drops from her in her first effort at defæcation. To prevent such an occurrence, I sometimes have placed a thin circular piece of cork upon the concave surface of the pessary, and then pass through it the two ends of a narrow white silk ribbon, a foot long, and through the hole in the pessary, which I afterwards tie. A loop will now hang out of the vulva, six inches long, through which I direct a strip of muslin to be passed, and afterwards to be tied around the waist of the patient. Thus secured, if it fall out it will not be lost.

It is directed by Dr. Dewees, that a few syringes, full of soap and water, be thrown up the vagina daily, to keep the parts clean. This is not always necessary, as, in most cases, washing the parts externally will be found sufficient.

The pessary will, occasionally, effect a radical cure, but in a majority of cases it only affords present relief.

The pessary neither interrupts sexual intercourse, nor prevents impregnation. In copulation, the male organ passes up between the anterior edge of the pessary and the arch of the pubis, and hence it does not interfere with the injection of the semen masculinum.

There are, however, objections to the use of this instrument, and some of a very serious character.

In the first place, it is very repugnant to the feelings of a lady of delicacy and refinement, to submit to the original introduction, and frequent replacing of the pessary by her physician.

2. When properly adjusted in the vagina, the posterior edge of the pessary rests in the posterior cul de sac of the vagina, and against the rectum, producing either irritation or constipation of the rectum; the anterior edge and part of the convex surface passes against the perineum, causing an increased, and often vitiated secretion.

3. The pessary produces not only costiveness, but hæmorrhoids, and, sometimes, stricture of the rectum.

4. It produces, sometimes, great irritation of the os tincæ and neck of the uterus, and produces a tendency to scirrhous and cancerous affections.

5. The uterus, by its own weight, and that of the intestines above it, causes the os uteri and a portion of the neck, occasionally, to enter the hole in the pessary, and there to become strangulated.

I attended a young and unmarried lady, a few years since, with prolapsus uteri.

In the course of the treatment, I introduced a small pessary, which, for a few months, gave her great comfort; but afterwards, complaining of some irritation, I made a per vaginam examination, when I found the os tincæ, and a portion of the neck, so tightly squeezed into the central aperture of the pessary, that it was with great difficulty that I could extricate them. This strangulation produced what is called the *irritable uterus*, for which this young lady was under treatment nearly two years, during most of which time her sufferings were intense. She, however, eventually recovered, and, I think, since married, and has one or more children.

The disease called irritable uterus is a rare, though one of the most formidable diseases I have ever met with. The best article that has ever been published upon that disease, may be found in a work entitled, "Gooch on the Diseases of Females."

In the year 1836, I was requested to attend a lady when she should require the assistance of an accoucheur. I called a few days after, when I learned that she was afflicted with prolapsus uteri, and was at that time wearing the pessary, and that she was in the fifth month of gestation. She said it was true she had not *quickened*, but she had all the other symptoms of pregnancy, such as suppression of the menses, morning sickness, enlargement of the abdomen, fulness of the breasts, &c.

I entertained some doubts with regard to her condition, and called again at the end of another month, when I found all her symptoms as before, only increased. She still had not quickened, although she was now in the sixth month of gestation. I told her I apprehended that she was mistaken with regard to her condition, and requested her to submit to a per vaginam examination, assuring her that if she was pregnant it was now time to remove the pessary, as it was useless at so advanced a period. She accordingly consented, and, upon examination, I found that the os tincæ had passed through the hole in the pessary, and the neck was strangulated by it. I ascertained, moreover, that the uterus was unimpregnated, and that all her sufferings arose from this irritation of the neck of the womb.

I advised this female to be confined chiefly to her bed for a few weeks, to be restricted in her diet, to have injections of cold flax-seed tea thrown up her vagina three or four times a day, and no longer to wear the pessary. She did so, and in the course of a month all irritation subsided, she again menstruated, and a few days afterwards was impregnated, carried the fœtus to the full period, when she presented her husband with a fine daughter, and in fifteen months more a healthy son. Whilst she wore the pessary she could not be impregnated, but as soon as it was laid aside, she became wonderfully prolific.

Although the wearing of the pessary does not always prevent impregnation, it is easy to imagine that, in this case, squeezed as the neck of the uterus was into the aperture of the pessary, and strangulated, conception was impossible, if the theory be true, that the semen must pass through the uterus and fallopian tubes, in order to fecundate the vesicle in the ovary.

THE UTERO-ABDOMINAL SUPPORTER.

Having now given a candid statement of the advantages and disadvantages which arise from the use of the pessary, I proceed to give my experience in the use of an apparatus, which, I am persuaded, meets all the indications in prolapsus uteri better than the pessary, and is not liable to so many objections; I allude to the *utero-abdominal supporter* of Dr. Hull.

Three years since, feeling satisfied, from extensive experience and observations, that something ought to be constructed that would better answer the purpose, in prolapsus uteri, than the pessary, I accidentally fell in with the *utero-abdominal supporter*, and tried it in a very troublesome case of prolapsus with the most delightful effect. The lady, previous to the time this apparatus was applied, rarely ventured in the streets on foot, so intense were her sufferings when she exercised her locomotive powers. But, by wearing this apparatus, she can walk for hours with little or no inconvenience.

This lady has now worn the *supporter* three years, and proclaims it to all her friends a "heavenly invention."

During the last two years, I have caused a large number of females afflicted with prolapsus to try the *supporter*, and am so much delighted with its success, that I can now recommend it to the profession with great confidence.

A medical friend of mine, in Chester county, too, has tried it in a few cases, with very satisfactory results.

This apparatus, which is worn externally, consists of a circular metallic band, about an inch wide, with a broad expansion behind and before, covered externally with leather, and internally with a soft muslin pad. Anteriorly, the pad is elastic, and broad enough to cover the whole hypogastrium, and the apparatus is so constructed as to make the pressure of this pad from before obliquely upwards and backwards. The pad, moreover, gives a congenial support to the weakened and relaxed abdominal muscles, takes the weight of the intestines off the fundus of the uterus, relieves "the dragging and bearing down sensations," which accompany prolapsus, and affords always temporary, and, sometimes, radical relief.

There is another part of this apparatus which consists of a strap that passes from the middle of the posterior broad expansion of the circular band between the limbs, and is attached to the broad expansion in front. Near the middle of this strap there is a sheath that receives a prism-shaped cushion or pad, made of compressed sponge, enveloped in linen. The whole apparatus somewhat resembles the T bandage.

The central strap, and prism-shaped pad meet a very important indication in the treatment. In all cases of long standing, the posterior part of the vagina and perineum become distended, so that there is a pouch-like projection between the posterior commissure and the anus. This projection is met by the prismatic or perineal pad, which soon obliterates the pouch, and restores the soft parts to their wonted tenacity. And if the case be a recent prolapsus, the pad will push up the perineum, and thereby diminish the calibre of the vagina, and elevate the displaced organ.

Some of the accoucheurs of this city refuse to recommend the *supporter* to their patients, contending that its use is contrary to sound philosophical principles.

This I deny, and hope I have already demonstrated that it is better adapted to meet all the indications, in prolapsus, than the pessary. Be this, however, as it may, I speak confidently as to the propriety of the remedy, because, in my hands, it has been very successful. The treatment that affords, in this displacement, either temporary or radical relief must be good, and that which is most successful is the best, even if it prove that some favourite theory has been erected upon a false foundation.

There is another supporter which operates upon the same principles, invented by a Mrs. Betts, a lady of intelligence and refinement, who resides at the corner of Third street and Germantown Road. This supporter has been in use in this city for about five years, but I have had no experience in its use until recently. I have, however, seven patients wearing it at present, with the happiest effect, and one with great comfort, who was not able to wear Dr. Hull's. This patient is very thin, and the spinous processes of her lumbar vertebræ unusually long, in consequence of which the iron band of Hull's supporter bruised her back, and so abraded the skin that she was obliged to discontinue its use.

My own opinion now is, that Mrs. Betts's apparatus is adapted to more cases than Dr. Hull's, yet I would give the preference to the latter, if the patient was very corpulent.

Mrs. Betts's apparatus differs from Hull's in having a broader belt to pass around the body, without the iron band being enclosed, and in a more broad, soft, and elastic perineal pad.

This she invented for herself; and as her case is as remarkable as it is interesting, and as it reflects credit upon the superior skill and sagacity of one of our medical brethren, I shall here give a brief sketch of it.

About fourteen years since, whilst residing in London, she had an abortion, by which she was reduced to a very low state of health. Her nervous system became greatly deranged, accompanied with palpitations of the heart, and, occasionally, violent attacks of spasmodic asthma. She was attended about nine years by the best physicians of London, without deriving from their treatment any benefit. They pronounced her complaint angina pectoris, in which the heart was especially involved.

Five years since, she sailed for the United States, and took up her abode in Philadelphia. Soon after her arrival, she consulted Dr. Malone, a respectable physician of the northern part of the city, who called Professor Jackson in consultation. After a thorough investigation of her case, the latter gentleman gave it as his judgment that the heart was free from disease, and the womb, which was prolapsed, was the seat of all her sufferings. This, she said, at once accorded with her own views, because she had often relieved herself of her most poignant sufferings by placing her two hands upon the lower part of her abdomen, just above the symphysis pubis, and pulling the intestines upwards and backwards—the very duty which her supporter is made so successfully to perform.

At this time, and under these circumstances, she invented for herself the supporter that she now offers to the public. She had not at that time either seen or heard of Dr. Hull's supporter, and is, therefore, entitled to the full credit of being the sole author of her invention.

The success of the supporter, in her case, is very remarkable. Since the day it was first applied she has never had a single paroxysm of asthma. By it, too, her nervous system was tranquillized; the palpitations of her heart assuaged, and her general health improved. And, although she had not had a child for eight years before, she conceived about two months after she began to wear the supporter, and at the full period she presented her husband with a fine son.

The *supporter* crept into use very slowly, but Mrs. Betts, at this time, receives as many as ten or twelve orders a week for them. She reports twelve cases that came under her own knowledge, in which her supporter succeeded after Hull's failed. She reports, too, some *radical cures*, which are sustained by the experience of Professor Jackson.

Mrs. Betts' apparatus, in my opinion, is best adapted to lean females, and Dr. Hull's to those that are fat and corpulent.

In, conclusion, the supporter is entitled to a preference, because the female, by wearing it, avoids the irritation of the vagina and the leucorrhœa, which the pessary almost invariably produces. Besides, the supporter offends less against female delicacy, as it does not require the hand of the physician to apply and carefully adjust it.

Again, if a young female indicates, by her symptoms, that she has prolapsus, and cannot overcome her repugnance to a per vaginam examination, the supporter may be tried. If she labour under the displacement, it will afford her relief; if she does not, it can do her no injury.

